# Understanding the complexity of existing fossil fuel power plant decarbonization

**DOI:** 10.1016/j.isci.2022.104758

**Published:** 2022-07-16

**Authors:** Chuan Zhang, Haibo Zhai, Liwei Cao, Xiang Li, Fangwei Cheng, Liqun Peng, Kangkang Tong, Jing Meng, Lei Yang, Xiaonan Wang

**Affiliations:** 1 Institute of Energy, Peking University, Beijing 100871, China; 2 Andlinger Center for Energy and the Environment, Princeton University, Princeton, NJ 08544, USA; 3 Department of Civil & Architectural Engineering, University of Wyoming, Laramie, WY 82071, USA; 4 Department of Engineering and Public Policy, Carnegie Mellon University, Pittsburgh, PA 15213, USA; 5 Department of Chemical Engineering and Biotechnology, University of Cambridge, Cambridge CB3 0AS, UK; 6 Princeton School of Public and International Affairs, Princeton University, Princeton, NJ 08544, USA; 7 China-UK Low Carbon College, Shanghai Jiao Tong University, Shanghai, 201308 China; 8 The Bartlett School of Sustainable Construction, University College London, London, WC1E 7HB, UK; 9 Department of Chemical Engineering, Tsinghua University, Beijing 100084, China

**Keywords:** Energy resources, Energy policy, Energy sustainability, Energy management, Energy Modelling, Energy flexibility

## Abstract

Growing national decarbonization commitments require rapid and deep reductions of carbon dioxide emissions from existing fossil-fuel power plants. Although retrofitting existing plants with carbon capture and storage or biomass has been discussed extensively, yet such options have failed to provide evident emission reductions at a global scale so far. Assessments of decarbonization technologies tend to focus on one specific option but omit its interactions with competing technologies and related sectors (e.g., water, food, and land use). Energy system models could mimic such inter-technological and inter-sectoral competition but often aggregate plant-level parameters without validation, as well as fleet-level inputs with large variability and uncertainty. To enhance the accuracy and reliability of top-down optimization models, bottom-up plant-level experience accumulation is of vital importance. Identifying sweet spots for plant-level pilot projects, overcoming the technical, financial, and social obstacles of early large-scale demonstration projects, incorporating equity into the transition, propagating the plant-level potential to generate fleet-level impacts represent some key complexity of existing fossil-fuel power plant decarbonization challenges that imposes the need for a serious re-evaluation of existing fossil fuel power plant abatement in energy transition.

## Graphical abstract

**Figure:**
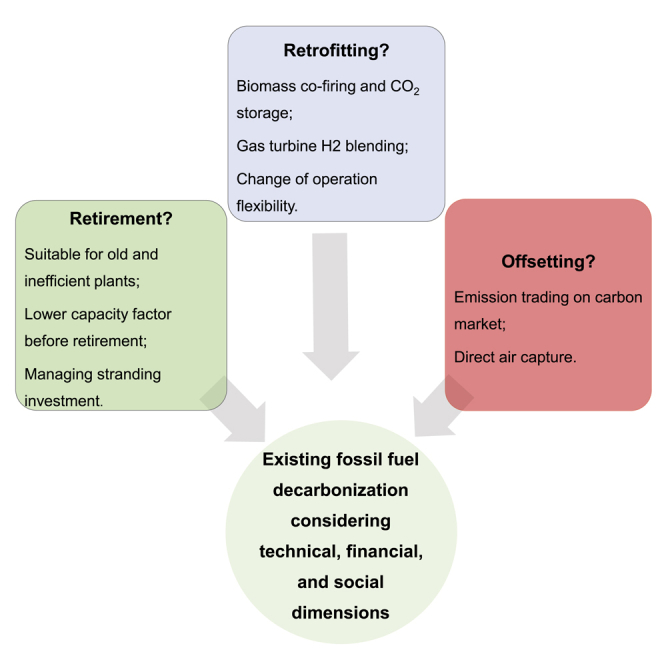

## Introduction

**Figure 1:**
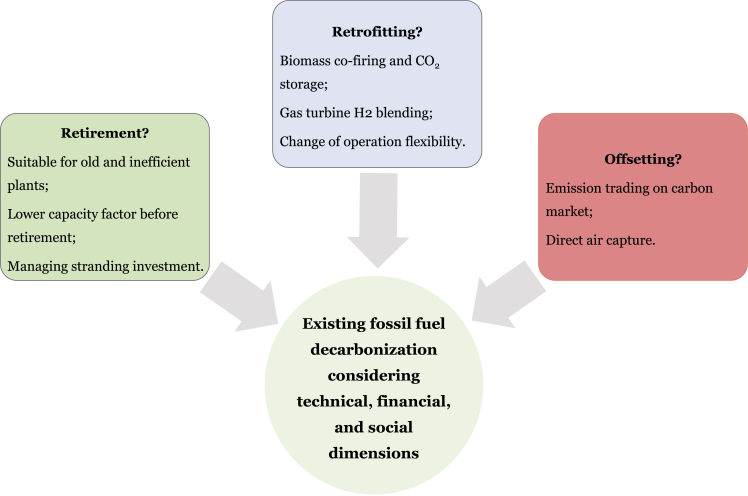
Different pathways for decarbonizing existing fossil fuel power plants, namely retirement, retrofitting, and offsetting Retirement could be achieved via deliberate planning; retrofitting could be achieved via biomass co-firing and/or carbon capture and storage retrofit; offsetting could be achieved via emission trading or external direct air capture.

Power generation is one of the largest anthropogenic carbon dioxides (CO_2_) emitting activities as industrialization and fossil fuels have dominated the power sector for decades. Climate awareness has been increasing greatly around the world, including ambitious targets aimed at an emission-free power sector by 2035 and a net-zero carbon economy by 2050 in the US, the emission peak by 2030 and carbon neutral by 2060 in China, and the net-zero carbon by 2070 in India. To achieve these targets, rapid and deep reductions of CO_2_ emissions from existing fossil fuel power plants to zero or quasi-zero level are required. Yet fossil fuel power plants are CO_2_-emitting infrastructure with high socio-technical inertia, which has resulted in high committed emissions or carbon lock-in (Grubert, 2020; Zhai, 2019). Recent studies show that committed CO_2_ emissions from existing fossil fuel power plants would be around 358Gt before 2050, whereas the latest IPCC report only suggests a remaining 420 Gt carbon budget in the 1.5°C scenario (Rogelj et al., 2018; Tong et al., 2019). As a result, such committed emissions from fossil fuels are high enough to jeopardize many other decarbonization efforts. On the other hand, recent high gas prices in Europe and power shortage in China have clearly shown that the pace and scale of fossil-fuel phase out should be carefully designed to secure the integrity of net-zero energy system transition (Carbon Brief Analysis, 2021; Carbon Brief, 2021; News, S.S., E&E, n.d.; Tollefson, 2018), as implied by changing “phase out” to “phase down” in COP26 (Wiebel et al., 2021). Major components of future deeply decarbonized power mix include variable renewable energy, decarbonized fossil fuel, and other clean firm generations like nuclear. The ratio between those resources can be optimized though, some cases report ultrahigh renewable penetration above 90%, whereas other cases rely more on decarbonized fossil fuel (Baik et al., 2021). Decarbonization of fossil fuel power plants could be achieved through early retirement, decarbonization retrofit, or emission offsetting (Figure 1). Emission offsetting provides an intermediate solution for curbing emissions from the easy-to-decarbonize sectors rather than a fundamental solution. Thus, this perspective mainly focuses on discussions on retirement and retrofitting. A systematic rethinking is strongly needed to address critical questions like which plants should retire, which plants should be retrofitted, how much the stranded investment is associated with retirement, what the required bioenergy supply and geological sequestration potential are with retrofitting, how emerging hydrogen (H_2_) economy could be integrated, and so on.

A large portion of existing studies has analyzed the technical feasibility and economics of fossil fuel power decarbonization at different temporal and spatial scales (Davis et al., 2018; He et al., 2016; Pehl et al., 2017; Xing et al., 2021). Generally, there exist two different approaches: one is fleet-level system modeling, whereas the other one is plant-level analysis. Fleet-level analysis typically relies on energy system modeling tools where the interaction between fossil fuel and renewables, nuclear, as well as energy efficiency and transmission, are explicitly simulated. Insights from such least-cost energy transition models could shade light on an ideal fossil fuel power transition pathway, and thus could serve as an important reference for policy making. Plant-level analysis focuses on one or more representative cases, using specific techno-economic models to analyze the impact of decarbonization retrofit on plant operation. Plant-level analysis usually takes in more detailed input parameters, thus resulting in more practical solutions for selected plants. Both approaches confirmed some untapped potential from fossil fuel power decarbonization, whereas fully acknowledging the remaining challenges with respect to technological, economic, environmental, and social dimensions of fossil fuel decarbonization. Moreover, it has been widely acknowledged that the integration of fossil fuel power decarbonization in a future energy system is influenced by a variety of techno-economic and policy uncertainties, for example fuel price, capacity factor, technology learning rate, and carbon tax. Thus, this perspective strives to highlight such complexity of existing fossil fuel power plant decarbonization and calls for a combined approach to handle such complexity, including plant-level pilot project design and fleet-level potential assessment from technical, financial, and social lens.

## Decarbonization pathways

**Figure 2:**
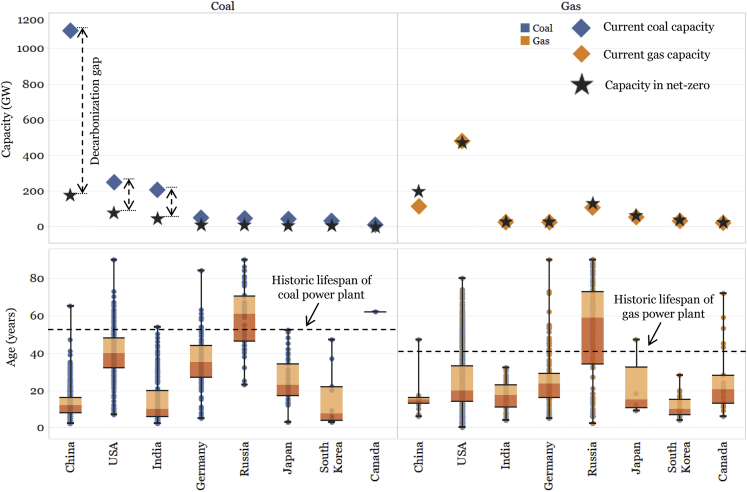
Gap between current fossil fuel power plant capacity and the targeted capacity in top CO_2_ emitting countries Also shown in this figure is the distribution of current coal and gas power plants compared to the historic life span of coal and gas power plants. The decarbonization gap between current capacity and target capacity in selected modeled scenarios, as well as the current lifespan and historic lifespan, highlights the importance of discussing “decarbonization of existing fossil fuel” is equally, if not more, important as “construction of new renewable.” (Source data based on global power plant database (Global Power Plant Database - Data | World Resources Institute, n.d.; Zhang et al., 2022), UN statistics (“UNdata, Electricity, net installed capacity of electric power plants, n.d.), and IEA (Net Zero Emissions by 2050 Scenario (NZE) – World Energy Model – Analysis, n.d.), see supporting information for details.).

*Retirement* of fossil fuel power plants has been discussed widely. Although massive coal phase out has happened in some developed countries, such as the US, UK, and Germany, it may be not as easy in developing countries. Firstly, most existing coal power plants are relatively old and inefficient in these developed countries (Figure 2) and their capital costs have been fully or largely amortized. In contrast, existing coal power plants are still young in most developing countries, which would slash the feasibility of early retirement (Cui et al., 2021; Zhai, 2019). For example, around 80% of China’s existing coal power capacity have operational age below 20 years (IEA, 2020). In such cases, early retirement of fossil fuel power plants could result in a considerable asset stranding effect, which is estimated at around one trillion US$ by 2050 to phase out around 2500GW existing fossil fuel power capacity (Saygin et al., 2019). In addition, the major replacement of retired coal power in many developed countries turns out to be natural gas rather than renewables. For example, in the UK, switching 15% of its generation mix (45TWh) from coal to natural gas in a single year achieves 25Mt CO_2_ reductions (Wilson and Staffell, 2018); similarly, in the US, replacing 188GW coal capacity with existing underutilized gas capacity would reduce CO_2_ emissions by up to 40% (Lafrancois, 2012). A large-scale shift from coal to natural gas for electricity generation, however, may raise some concerns: firstly, the climate benefits of the shift could be partially, or even fully, eliminated by the methane leakage from natural gas production, which needs to be further quantified (Brandt et al., 2014; Perkins, 2019); secondly, there is still debate about the future role of natural gas in an emission-free generation portfolio. Some argue that building new unabated natural gas capacity is not a robust long-term strategy, whereas others consider flexible gas generation with carbon capture and storage (CCS) as an important clean firm power source in future variable renewable energy dominated power system (Busch and Gimon, 2014; Mac Kinnon et al., 2018; Zhang et al., 2016). Regarding early retirement, the difference between stranded capacity (per MW), stranded generation (per MWh), and stranded investment (per $) need to be differentiated, current discussions tend to focus on stranded capacity while omitting the fact that fossil fuel power plants are OPEX-intensive instead of CAPEX intensive, thus if fossil fuel power plants can actively lower their capacity factor through overcoming flexibility issues and getting market incentive, there would be only stranded generation, not stranded capacity or stranded investment. Thus, a win-win situation where fossil fuel and renewable co-evolve would be possible for partial decarbonization targets. In the long run, for net-zero decarbonization targets, exploring the possibility of decarbonizing existing gas power plants through CCS becomes important.

*Biomass* utilization in power generation is considered carbon-neutral owing to the atmospheric CO_2_ removal capability of biomass. Biomass co-firing in coal power plants (up to 30%) has been proved possible without largely modifying the existing infrastructure (Wang et al., 2021; Xing et al., 2021). Based on the latest IPCC estimation a 100EJ annual primary energy generation from biomass potential could be expected (AR6 Climate Change, 2022, n.d.), however, the debate around biomass food-energy-environment trilemma has not stopped over the past decades (Heimann, 2019; Sayed et al., 2021; Tilman et al., 2009). Although successful case studies on biomass utilization have been reported and biomass utilization pathways have been designed globally (Goldstein et al., 2012; Staples et al., 2017; Yang et al., 2021), the broad environmental impacts (water and land use, soil erosion, natural wildlife, and so forth) of large-scale biomass deployment, in particular dedicated biomass, should be further assessed and carefully taken into the decision making related to the cultivation, collection, and transportation process of biomass (Chang et al., 2017; Chaudhary et al., 2015; de Jong et al., 2019; Hof et al., 2018; Rowe et al., 2009), the low energy efficiency of biomass conversion into usable energy and the inherent properties of biomass (e.g., lower heating value, higher moisture content, and lower grindability) also undermine the large-scale integration of biomass (Yang et al., 2021). Moreover, optimal allocation of biomass resources between different end users (e.g., electricity, heat, transportation fuel, as well as food and feed) is a non-trivial problem that needs global optimization with many case-dependent parameters, including geographical distribution of biomass supply. A recent analysis shows that when considering all these together, biomass could account for 5500TWh power supply for 2050 in IPCC’s 2°C scenario (Staples et al., 2017); however, the cost-effectiveness and ecological impact of such biomass deployment have not yet been evaluated extensively. Moreover, the results of the most least-cost energy system models do not favor dedicated biomass utilization in the power sector compared to the fuel sector. In the case of the US national energy supply, less than 2EJ of biomass, or around 1/6 of total available biomass assumed in the model (Jenkins et al., 2021; Larson et al., 2020), is used in the power sector, whereas the majority is used in the fuel sector through biomass gasification and pyrolysis with CCS owing to the general fact that the decarbonization of fuel production is more difficult than the decarbonization of power production.

*CCS* is an integrated process of CO_2_ capture, transport, and storage to prevent CO_2_ release into the atmosphere. Although CCS could have a unique role in reducing the carbon intensity of fossil fuel-fired power and industrial systems, especially in scenarios with long-term horizons, it must be noted that the rhetoric on CCS has not been turned into large reality so far (Bui et al., 2018; “CCUS in clean energy transitions,” 2020; Greig and Uden, 2021); more projects such as Petra Nova and Boundary Dam are needed to upscale the mega-scale CCS capacity to giga-scale for material climate benefits (CCUS in Power – Analysis, n.d.). Even though the technology readiness level of power plant post-combustion CCS has already reached the commercial level, CCS integration into a typical coal power plant would induce at least a 50% increase in the generation costs, thus increasing the generation cost of fossil fuel power plants in competitive electricity markets (Van Den Broek et al., 2009). Enhanced oil recovery, namely the draining of oil wells through the sequestration of carbon dioxide, could provide additional incentives for CCS deployment. However, upscaling of CCS from megaton to gigaton to produce material climate change mitigation effects still lacks enough incentive as a consequence of low carbon price and technology learning rate (Benson and Deutch, 2018; Kolster et al., 2017). Additional prospect for CCS comes from the innovation in power cycles, such as Allam cycle, which could significantly reduce the cost of CO_2_ capture through the integrated design of oxy-fuel combustion and supercritical CO_2_ turbine. Facing challenges similar to the biomass supply curve, the cost curve of CO_2_ storage is also widely varied in literature. In the case of the US, the annual storage potential changes from 1Gt to 3Gt with storage cost ranging from −10$/ton to $70$/ton (Jenkins et al., 2021). Many studies argue CCS development would not be constrained by the lack of underground storage capacity. But, there is also plenty of criticism on such a conundrum around CCS (Lane et al., 2021). Deep CCS beyond 90% CO_2_ capture could be another technology option for the “last-mile” problem in decarbonization. In certain situations, it provides a lower marginal decarbonization cost than direct air capture (DAC) (Dods et al., 2021). Coupling biomass with CCS, known as BECCS, is a negative emission technology (NET), which is included in many mitigation pathways. Opinions around BECCS are also polarized: although many studies treat BECCS as an effective technology to offset carbon emission overshoot, others argue that the incorporation of BECCS and other NETs into the mitigation pathway could postpone the deployment of non-biomass renewables, thus resulting in risky carbon lock-in. Similarly, the gap between the required deployment scale and current demonstration projects states a clear urgency for upscaling (Hanssen et al., 2020; Lenzi et al., 2018; Van Vuuren et al., 2017; Xing et al., 2021).

The role of existing fossil fuel power plant decarbonization also comes from its interaction with other low-carbon generation technologies (e.g., nuclear, wind, solar, and hydro). The rapid decrease in the levelized cost of electricity (LCOE) of solar and wind has made renewables the cheapest power source in many places around the world, predictions falling behind of such rapid technology cost reduction have been recognized as the main reason for the failure of energy system models. In addition to such progressive technology advancement, disruptive technologies, like the fourth generation of nuclear, could also largely impact the energy supply mix. Many efforts in recent studies have pointed out that clean firm power generation, including natural gas with CCS, could significantly reduce the average cost of electricity in ultrahigh renewable penetration cases above 90% (Baik et al., 2021; Sepulveda et al., 2018). In light of such a comparison, exploration of decarbonized fossil fuel power generation bears some significance in the long term.

## Complexity

**Figure 3:**
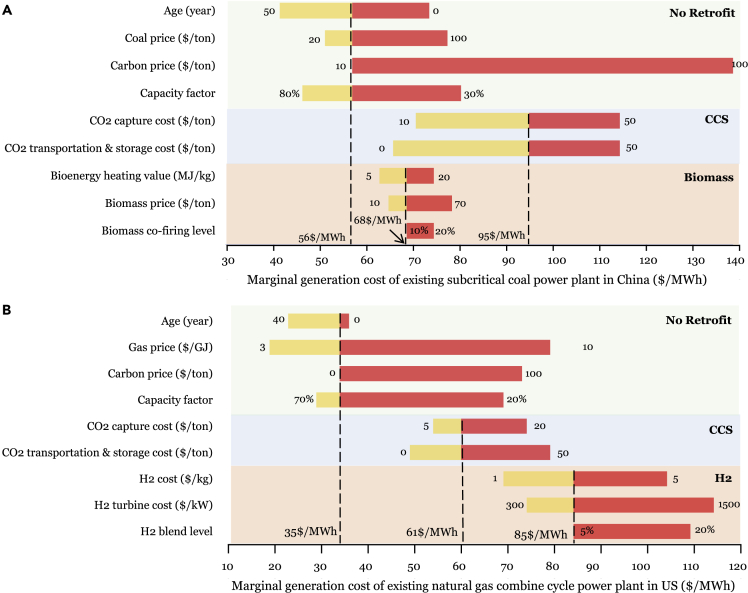
Sensitivity of existing fossil fuel power plant generation cost to different techno-economic factors This figure demonstrates the effect of various factors, including power plant age, fuel price, carbon price, CCS cost, biomass price, H_2_ cost, and capacity factor on the marginal generation cost of (a) a typical PC power plant in China and (b) a typical NGCC power plant in US. Shown in this figure is the operation cost of existing fossil fuel power plants depending on case-dependent parameters, which illustrate the complexity of existing fossil fuel decarbonization (See supporting information for details).

To further illustrate how the interplay of various uncertainties could influence the generation cost of existing fossil fuel power plant, single-factor sensitivity analysis is conducted for a standard subcritical pulverized coal (PC) and a natural gas combined cycle (NGCC) power plant (Cui et al., 2021; Guo et al., 2016), which are two representative configurations in current fossil fuel generation fleet. Four different scenarios are investigated, including a reference scenario without any retrofit, a CCS retrofit scenario where a fossil fuel power plant with CCS is built, a biomass retrofit scenario where biomass co-firing is considered for a PC plant and an H_2_ scenario where H_2_ blending is considered for NGCC plant, respectively (Díaz-Herrera et al., 2021; du Toit et al., 2020). Given future uncertainty related to such technologies from either lack of enough demonstration projects (in the case of CCS and biomass) or current technological bottlenecks (in the case of H_2_), major model inputs are changed across a wide range of uncertainty on the base values (see Figure 3 and supporting information for details). Single-factor sensitivity analysis for PC and NGCC power plants shows that the generation cost could change from the nominal 56$/MWh to 40-140$/MWh, and from the nominal 30$/MWh to 20-115$/MWh, respectively. Such results clearly denote that the cost of retrofitting existing fossil fuel power plants is a complex function of various factors, including but not limited to CCS cost, H_2_ cost, biomass price, fuel price, and carbon price, and so forth. Again, understanding the complexity of existing fossil fuel power decarbonization has important implications for future scenarios. For example, as renewable penetration increases, the role of fossil fuel power plants in a generation mix would transform from a baseload provider to a following-load provider and/or operating reserves, which would result in a lower capacity factor. Sequentially, the external incentives (e.g., revenue from the capacity market) needed to drive decarbonization integration would increase. Such results require policy makers to deliberately consider trade-offs between “adding new renewables” and “subtracting existing fossil fuel.” Otherwise, a loss-lose situation, in which unabated fossil fuel power plants continue to emit a large amount of CO_2_ and renewable generation is curtailed, could occur (Fouquet, 2016).

In addition to the aforementioned techno-economic factors, there are also socio-political dimensions that should be considered for existing fossil fuel power plant decarbonization (Selosse and Ricci, 2017; Sun et al., 2021). It is a dilemma that the number of actual operational pilot retrofit projects is lagging far behind the scale required for deep decarbonization; as a result, not enough large-scale experience is available to jumpstart the learning-by-doing process and then iteratively accelerate the “research-development-demonstration” cycle toward widespread implementation. Such a lack of bottom-up experience accumulation accounts for the main reason why certain key modeling parameters like capture cost diverges across different studies (Anadón et al., 2017). As a result, some future technology projections in top-down energy system models come from expert elicitation with bias, thus undermining the credibility of energy system models (Meng et al., 2021). Moreover, capital investment in power plant retrofitting is usually very large. Under current situations, the risk of sunk investment and the unclear scope of green investment are all barriers to investment in retrofit projects for decarbonizing existing fossil fuel power plants (Ball et al., 2021). Public finance institutions, green banks, multilateral banks, and national development banks could play an important role in such project development. Treating biomass or CCS retrofit projects as a green investment option becomes important to motivate financial support for such technologies. Increasing global carbon price could be critical in driving the uptake of such retrofit projects, as previous studies suggest, carbon pricing in conjunction with other incentives (such as tax credit for carbon sequestration in the US) remains one of the most effective manners to drive existing power plants decarbonization in China (Bertram et al., 2015; Mo et al., 2021). However, the decarbonization of the existing power sector has more technology options other than existing fossil fuel retrofit, if pilot decarbonization projects increase to a large enough scale to accelerate the learning-by-doing curve, it could stand out from other competing technologies such as VRE with storage, nuclear, and H_2_; on the other hand, failure to materialize the retrofit decarbonization potential could also happen. Besides, the action time definitely matters for such existing fossil fuel power plant decarbonization. Most decarbonization pathways require net-zero power generation by no later than 2050. However, a typical project timeline for such retrofit projects takes years from planning to finishing, such urgency of timing action further blurs the prospect of the front-end time spent on demonstration project establishment and large-scale follow-up projects.

## Just transition

The transition to net-zero provides both opportunities and challenges to increase universal justice in the energy sector (Peng et al., 2021). The current fossil-fuel-dominated power sector is not considered as just in the following aspects: (a) the “polluters-pay” principle has not been well followed, which means the large fossil fuel companies have not taken full responsibility for the pollution and greenhouse gas emission externality from their operations; (b) the energy bills for the poor are unproportionally large compared to the rich, which undermines the basic product characteristics of electricity. How to ensure such marginalized people are positively impacted during the fossil fuel decarbonization transition is the grand challenge of just transition (Perlaviciute et al., 2021). The first and most important aspect of just transition is to ensure electricity access at a low energy price, especially for the poor to avoid energy poverty (Carley and Konisky, 2020; Frankowski and Tirado Herrero, 2021). If the ongoing net-zero energy transition undermines poverty alleviation (listed as first of the 17 United Nation Sustainable Development Goal), it should not be perceived as just. One of the biggest reasons why divorcing fossil fuel power plants quickly is very difficult is the fact: in many places, fossil fuels are still considered the cheapest energy source by central or local government, which may or may not be true according to the specific political-economical condition (Baker et al., 2021; Murtagh et al., 2021; Perlaviciute et al., 2021). Gradual fossil fuel subsidy reform plus stable renewable energy subsidies could narrow the macroeconomic impact of phasing out fossil fuel subsidies, whereas the impact differs among different countries and rich/poor people. The general impact would be larger for subsidizing, energy-exporting countries while smaller for non-subsidizing, energy-importing countries (Jewell et al., 2018; Ouyang and Lin, 2014). The justification of fossil fuel subsidies as a manner to promote energy access to low-income people needs to be re-designed if fossil fuel subsidy is reformed (Couharde and Mouhoud, 2020). If divestments from fossil fuel could be pooled into the investment of clean energy, a smooth transition from fossil fuel to renewable could be accelerated (Andrijevic et al., 2020). Raising the required money from both public and private sectors should be considered. The possibility of combining such costs with COVID-19 stimulus plan should be explored.

The second dimension of just transition relates to fossil fuel-associated employment. The first barrier here is to fully understand the current employment data related to the fossil fuel industry supply chain (mining, transportation, power generation, waste treatment). Such data are often outdated and could not reflect the current situation of fossil fuel sector employment (Burke et al., 2019). For example, the coal-related jobs have already dropped a lot even without coal phase-out owing to the increasing automation and machine intelligence (Paredes and Fleming-Muñoz, 2021). The distribution of education level and skill set is also important when coming to fossil fuel-related employment. In the case of employment transfer, such information would be vital to decide where such impacted people should go. Early studies have been conducted in some developed countries. However, it is more difficult for developing countries owing to the lack of data infrastructure; the expenses on such employment transfer and labor retraining should be counted into the cost of transition (He et al., 2020; McCauley et al., 2019).

The last dimension of just transition comes from the co-benefits of decarbonization in air quality and public health. Although it is widely acknowledged that existing fossil fuel power plant decarbonization retrofit would bring significant air quality co-benefits, yet it is also true, and sometimes can be omitted, that for people without access to affordable energy, the air quality benefits can be neglected when it comes to personal energy decision-making (Barrington-Leigh et al., 2019). Such individual interests would be reflected in institutional performance and country ideology, which constitutes a large reason for the so-called carbon lock-in. To some degree, the experience from the “first pollute/emit, then clean/decarbonize” development model is so successful and familiar to energy decision makers that more universal education and stringent climate policy are needed to gradually switch the conception here. In summary, air quality co-benefits must be evaluated in the context of affordable and reliable energy access. Otherwise, it will not make sense regarding equity.

Putting these all together, just transition must be considered during the existing fossil fuel power plant decarbonization (Krumm et al., 2022). In fact, there is a different perception of fairness among different countries, regions, companies, communities, and individuals, which makes it difficult to produce a “everyone is satisfied” case. In the specific case of existing fossil fuel plant retrofitting, there will be frontrunners with a higher risk. Future possibilities where early investments are not rewarded could happen, such financial and social risks should be properly insured to motivate first movers, rather than making it a race to be second, or even doing nothing but wait if there is room for existing fossil fuel decarbonization in the future (Krumm et al., 2022).

## Policy implications

Net-zero energy systems require a carbon-free power sector, among which unabated fossil fuel use will be largely reduced in the long run. However, the pace and scale of fossil fuel phase-out or phase-down must be carefully planned to balance the trade-off between energy security in the short term and energy sustainability in the long term. At a high level, this article strives to highlight such complexity of existing fossil fuel power plant decarbonization and call for a combined approach to handle such complexity including plant-level pilot project design and fleet-level potential assessment from technical, financial, and social perspectives (Cherp et al., 2018).

Developed and developing countries face different situations when it comes to existing fossil fuel power plant decarbonization. Retiring old and inefficient plants in developed countries seems to be a straightforward solution. In contrast, in developing countries, many plants are still quite new, and the investment has not been fully amortized. Thus, early retirement faces stranded investment risk that remains to be mitigated. For relatively new fossil fuel power plants, the difference between stranded capacity and stranded generation has to be highlighted. Through actively lowering operation capacity factor, potentially facilitated by electricity market incentives, existing fossil fuel plants could play the important role in residual load supply in a variable renewable dominating power system. Retrofitting technologies including biomass and/or CCS bears more risk. At a plant level, there is still limited experience learned from pilot projects to derive valid cost information for general models, which is viewed as the first uncertainty. Uncertainty also exists at a fleet level around biomass supply potential, geological carbon storage potential, among other factors that could impact the overall decarbonization potential. All these compose the technical complexity of existing fossil fuel power plant decarbonization. The complexity of existing fossil fuel decarbonization is further elevated by non-technical dimensions, including financial investment mobilization and employment impact. Regarding these dimensions, just transition has come into the discussion for a while, yet a transparent and auditable accounting of fossil fuel industry employment is still missing in many countries. Also, we argue that the widely acknowledged air quality co-benefits have to be more effectively considered for marginalized people with limited energy access in poor countries.

From a macro perspective, a combination of top-down energy system optimization with bottom-up demonstration project design is strongly needed. Through top-down systematic optimization, optimal resource allocation in and out of the energy sector could be made in economy-wide decarbonization; through bottom-up demonstration projects development, quantification of key uncertainties around major energy technologies could be achieved. There is not a “one-size-fit-all” solution for such existing fossil fuel decarbonization problem, making global planning at the fleet level is needed and local exploration at the plant level is equally important. A positive cyclical iteration between globally designed and locally adapted solutions could be established to catalyze the upscale of existing fossil fuel decarbonization retrofits to produce evident emission reductions (Table 1) (Pye et al., 2021; Zhang et al., 2022).

**Table 1:** A list of technical, financial, and social enablers/barriers to existing fossil fuel decarbonization

Decarbonization pathways	Key enablers and barriers
Retirement	• *Technical:* The capacity, age, and energy efficiency of operating power plant, possibility of operating such plants at lower capacity factor • *Financial:* Stranded investment solution, whether all investment has been vested or not, if not who will compensate the unvested capital and how to do it • *Social:* Local community reactions to the retirement of existing power plants, how such an impacted community is managed in terms of employment transfer, and so forth. Learning from both successful and unsuccessful retirement lessons to create a triple-win situation for plant owner, fossil fuel community, and energy consumers.
Biomass	• *Technical:* Supply curve of major sources of biomass (agriculture residue, forest residue, waste, dedicated energy crop, and so forth) and related supply curve, optimal biomass utilization through co-firing, gasification, pyrolysis, and so forth. • *Financial:* Transportation cost of biomass (ship, truck, rail), investment portfolio of biomass retrofit projects, and related financial risks • *Social:* Monitoring the short-term and long-term environmental and ecological impact of large-scale biomass harvesting from various channels Learning from biomass utilization experience and aggregating the plant level potential to the fleet level to identify the upper and lower limits of biomass enabled decarbonization potential and corresponding cost.
CCS	• *Technical:* Enablers of fossil fuel power plant CCS (e.g., boiler type, plant age, efficiency, and post-combustion cleaning), impact on plant operation flexibility • *Financial:* Generation cost (e.g., levelized cost of electricity) before and after CCS is retrofitted, capital investment for project development, and experience with financial risk management • *Social:* Social acceptance of geological storage site from technical, economic, social perspectives, monitoring short-term and long-term stored CO_2_ re-emission and other issues Learning from early CCS demonstration projects from both power and other sectors, setting up the required storage potential, capital investment for retrofits, and estimating the fleet-level potential aggregated from the plant level.

## Limitations of the study

Finally, it must be noted that although our analysis focuses on existing fossil fuel decarbonization, a systematic assessment of the interaction between fossil fuel decarbonization, variable renewable, clean-firm generation, transmission, energy efficiency, and beyond in a net-zero world has to be conducted to determine their optimal pathways. Also, there are studies showing retrofitting existing fossil fuel plants could be much less feasible and economic than building new plants (Rohlfs and Madlener, 2013; Rubin et al., 2015, p. 2), in particular for CCS. In such cases, our discussions on existing fossil fuel decarbonization apply to the condition that fossil fuel power plants are already built, whereas urgent emission reductions are critically needed to achieving net-zero no later than 2050 regardless of new fossil fuel with CCS or not. By no means, it represents a future that we can continue to build new business-*as*-usual fossil fuel power plants and bet on later decarbonization retrofits.
